# Diseases of the Vault of the Pharynx

**Published:** 1887-01

**Authors:** H. Gradle

**Affiliations:** Central Music Hall


### ON CERTAIN DISEASES OF THE VAULT OF THE PHARYNX.

*By* H. GRADLE, M.D.

[Read before the Chicago Medical Society, December 6th, 1886.]

METHODS OF EXAMINATION. HYPERTROPHY OF THE PHARYNGEAL TONSIL. IRRITATING PLUGS IN THE CRYPTS OF THE TONSIL. HYPERTROPHIC AND SIMPLE CATARRH. THE BURSA PHARYNGEA.

There exist various diseases of the naso-pharyngeal space, which are but too often overlooked by the general practitioner, or, being simply termed catarrh, are treated in a routine and inefficient manner. The frequency of these complaints is shown by the large number of persons in this climate, whose snuffling and hawking and clearing the throat indicates the annoyance they feel in the pharynx. While these affections are indeed not a serious matter ordinarily, the frequency with which ear disease involves both ears at the same time is the best proof of the importance of the space where the two Eustachian tubes meet. Far the most important aspect of pharyngeal anomalies is their liability to lead to catarrhal or suppurative inflammation of the ears, and from this point of view I have mostly seen my patients.

The difficulty of examining the upper part of the pharynx—often overestimated—is reduced by the use of cocaine in persons with sensitive throats and those having imperfect control over their palatal and pharyngeal muscles. The rhinoscopic view is much more comprehensive and not more difficult to obtain, if we employ a large mirror up to one inch in diameter. Where a small mirror can be used a larger one is generally tolerated as well. Where the space between the soft palate and the rear pharyngeal wall is limited, the examination is scarcely satisfactory without a palate look. I prefer one made of wire in the form of a loop, curved on the flat, and find this of but very little inconvenience to the patient. But like all other modes of medical examination, posterior rhinoscopy requires some practice before success can be attained. On an average I have succeeded in gaining a fair view in three patients out of four at the first trial and of the remainder one-half can train themselves until they become tolerant. A small number of people never learn to submit. Children old enough not to be frightened are no more difficult to examine than adults.

The morbid condition which I shall principally deal with in this paper, viz., enlargement of the pharyngeal tonsil, can be recognized without the mirror, by introducing the finger through the mouth behind the soft palate and feeling whether the space is limited by a smooth roof or whether it is filled with soft yielding growths. The rear end of the inferior turbinated bones must not be mistaken for growths. This examination is quite disagreeable to the patient.

The pharyngeal tonsil is a structure insufficiently described or even ignored in many text-books. It forms a mass of lymphoid glandular tissue lodged at the roof of the pharynx, with very delicate flat extension down the sides of the pharynx up to and partly into the Eustachian orifices. Like the oral tonsils, this gland is frequently subject to hypertrophy, and for reasons equally unknown. The trouble occurs perhaps more often in scrofulous children than in others. This hypertrophy impedes the passage of air through the nose, especially when a temporary congestion is superadded by reason of a fresh attack of catarrh or simply from the low position of the head during sleep, for the tonsils contain enough vascular tissue to be capable of variations in size and may be sometimes seen to shrink very considerably from the application of cocaine, though only for the time. The filling up of the post-nasal space forces the patients, usually children, to sleep with their mouths open and accordingly they snore, sometimes to an annoying extent. The voice has a "dead" sound, the nasal consonants n, m and ng being indistinct and resembling d. The voice is oftentimes sufficiently characteristic to reveal the disease to an expert. Besides impeded nasal respiration a venous congestion is the inevitable result of enlargement of the pharyngeal tonsil from pressure upon the lateral walls of the pharynx beneath which the return veins pass along. This venous congestion is manifested by the dark rings around the eyes, and dilated veins sometimes seen on the conjunctiva, and the thickened lips often found in scrofulous children, all of which recede after extirpation of the gland. Besides, the enlarged tonsil may give rise to reflex disturbances, frequent headaches of the occipital type and sometimes persistent cough, accompanied or not by bronchitis. A striking case was that of a ten months' babe under my charge for ear disease which had suffered for many weeks from a chronic bronchitis, according to the diagnosis of a competent physician. On finding the suppuration of the ear unusually tedious I explored the pharynx, detected the enlargement of the tonsil and immediately removed that gland with the curette. Within one day all signs of bronchitis ceased. Several of the children that I have seen with this trouble were subject to night-mare and sometimes night-terror. In one case this amounted to transient delirium during several weeks. The boy, at the time twelve years old, awoke almost every night frightened and unable to comprehend the situation. The cause, as the mother could judge from his actions, seemed to be a want of breath. All of these reflex symptoms as well as the impediment to nasal respiration are permanently removed by extirpation of the gland.

The most serious aspect of hypertrophy of the pharyngeal tonsil is the liability to ear disease. This may be catarrhal or suppurative and is always persistent and apt to relapse, if temporarily cured, as long as the hypertrophy remains. But after removal of the tonsil the inflammation of the ear can be easily managed.

The enlargement of the pharyngeal tonsil is commonly referred to in the literature under the name of adenoid vegetations. This name is justified, whenever coxcomb-shaped projections hang down, giving the mass somewhat the appearance of an inverted head of cauliflower. These separate prominences on the surface of the tonsil result from the natural divisions of the normal structure into ridges. The most common form in which I have seen hypertrophy of the pharyngeal tonsil in this country is, however, not that of separate vegetations, but the entire gland appears uniformly enlarged into a more or less hemispherical body with smooth surface, filling out the naso-pharyngeal space. It has seemed to me as if this more diffused form of hypertrophy were more common in older than in younger children. The enlargement may be of a soft or of a firm texture, the former predisposing to frequent hemorrhages from the nose and pharynx. It seems that this enlargement recedes to a certain degree, if not completely, as the period of growth is passed, for in adults the pharyngeal tonsil is rarely found enlarged to any extent.

Hypertrophy of the pharyngeal tonsil cannot be treated successfully to-day except by surgical means. The galvano-cautery may be used, passing a suitably curved electrode with flat platinum tip up behind the soft palate and thrusting it into the enlarged gland. This must be repeated quite often, unless we make each sitting more thorough by gouging as much as possible with the platinum blade, while it is in the midst of the tissue. After the thorough application of a spray of 4 per centum solution of cocaine the pain is not very great, especially if we wait about fifteen minutes before burning. More satisfactory than the galvano-cautery is the use of a sharp curette, especially in the form recommended by Trautmann (*Anat. Path. & Klin. Studien ueber Hyperplasie d. Rachentonsille*, Berlin, 1886). This consists of a stout sharp spoon from 11 to 15 millimetres in diameter and not perforated. About 25 millimetres from the end the shank is bent to an angle of 150°. The patient being securely seated, the curette is passed through the mouth up behind the soft palate, pulled forward so as to glide up between the edge of the vomer and the pendent glandular mass, whereupon it is pushed backward and downward. Subsequently the cutting is completed with the sharp lateral part of the edge and, if possible, the removed mass is brought out in the concavity of the scoop. The hemorrhage is sometimes copious at the first moment, but I have never seen it alarming. It can always be controlled by gargling with ice water. As soon as the flow of blood is stanched the curette is again introduced until the entire mass is removed or the patient refuses to submit any longer for the time being. Since the gland glides away from the edge of the curette it always requires three or four attempts before the extirpation is complete and in sensitive patients often four or five sittings may be necessary. I have found it of advantage to use, besides the Trautmann sharp spoon, several other forms of scoops. These are not a mere sharp spoon, but a hollow scoop and bent so that they can be introduced along the sides of the pharynx and gouge their way through the tonsil from either the right or the left side. After the sitting is over we can decide how complete the result has been only by means of the finger, for as a rule the oozing of blood prevents the use of the mirror. Although this operation is by no means neat, and often appears bungling, it is really not very painful and I have never been forced to postpone a second operative attempt upon a child. Moreover, the result is in all cases exceedingly gratifying, be it done in the interest of the ears or simply for the removal of the impediment to respiration. My experience amounts to nearly thirty cases upon which I have operated, while about half of that number have declined or postponed operative interference. No after treatment is necessary.

The ring-shaped knife which Meyer, who first described adenoid vegetation, passes through the nose is, according to my experience, more unpleasant to the patient than the scoop introduced from the mouth. The galvano-caustic loop is very difficult to apply, and impracticable where there are no separate vegetations.

The pharyngeal tonsil may be a source of irritation, even when not enlarged to any extent. Like the oral tonsils it contains crypts which are sometimes filled with white plugs consisting of masses of bacteria. I have seen at least six or eight patients in whom pharyngeal annoyance and ear disease could be traced to this condition as the starting point. In these cases destruction of these crypts with the galvano-cautery proved a sufficient treatment. The diagnosis, of course, could be made only with a rhinoscopic mirror.

The adenoid tissue continuous with the pharyngeal tonsil forms in some persons thickened red spots at the upper part or the lateral walls of the pharynx above and behind the Eustachian orifices. These irritated follicles have seemed to me to exert an unfavorable influence upon the ears in several instances, for after their galvano-caustic destruction the catarrh of the middle ears proved more manageable than it had been prior to this operation.

Without wishing to discuss at any length naso-pharyngeal catarrh, I think it worth while to call your attention to the difference between the hypertrophic form of this disease and the simple catarrh not accompanied by any visible lesions. The form in which the pharynx appears uniformly red and thickened yields quite readily to treatment. According to my experience nitrate of silver has given better results than any other remedy. I have made use of it either as a four per centum wash applied with a brush, or preferably in the form of a dry powder mixed with six times its weight of talc and blown in with an insufflator. I find, however, the most agreeable mode to the patient is the use of the four per centum spray, but this is awkward to the physician on account of the liability to stain the clothes.

Of all pharyngeal diseases I know of none more rebellious to treatment than the simple catarrh without visible lesions. The presence of follicles on the posterior wall of the pharynx does not give a better prognosis, for I have found treatment applied to scattered follicles in the form of astringents, or the galvano-cautery, almost inert. Where nothing can be seen on inspection, but the patient complains of discomfort and the presence of mucus and suffers besides from catarrhal disease of the ears, almost every mode of treatment has been unsatisfactory in my hands as far as the pharynx is concerned, although the aural symptoms may be modified or removed.

Tornwaldt (*Ueber die Bedeutung d. Bursa Pharyngea*, etc., 1885) has attributed this form of naso-pharyngeal catarrh without visible lesions to disease of what he calls the *bursa pharyngea*, a pit or recess of the mucous membrane at the roof of the pharynx in the rear part of the center of the pharyngeal tonsil. The pit which he describes as the entrance to this bursa I have seen with a large rhinoscopic mirror many times, but by no means in all persons. He claims to demonstrate the existence of catarrh in this recess by the formation of crusts around the opening, or the oozing of pus or mucus from it while under examination. This condition I have never been able to find. I am therefore skeptical as to the existence of the normal bursa pharyngea and entirely distrustful as to catarrh limited to this recess. I have, however, followed Tornwaldt's suggestion and cauterized this region with the galvano-cautery, even though no local appearance pointed to it as the source of the pharyngeal discomfort and disease. The results have not been uniform; in some cases they have been entirely negative, while in about one-half of the dozen patients on whom I have tried this method some amelioration was obtained. Tornwaldt also describes cysts as the result of closure of this bursa. These cysts at the roof of the pharynx seem to be rare in this country; I have only seen them twice and I am more inclined to consider them originating in the tissue of the pharyngeal tonsil than from closure of the alleged bursa. The plain indication is to open these cysts and to destroy their cavity with the galvano-cautery. The results of this treatment were satisfactory to the patients.

Central Music Hall.

